# Supplementary material for: Fast joint parity measurement via collective interactions induced by stimulated emission
Source: Nat Commun. 2024 Apr 8;15:3045. doi: 10.1038/s41467-024-47379-1 (PMC11001884; doi:10.1038/s41467-024-47379-1)
Supplement: Supplementary file 1 — Supplementary Information [file 41467_2024_47379_MOESM1_ESM.pdf]

Supplementary Materials: Fast joint parity measurement via collective interactions induced by stimulated emission

Sainan Huai,[1,2] Kunliang Bu,[1,2] Xiu Gu,[1,2,*] Zhenxing Zhang,[1] Shuoming An,[1] Xiaopei Yang,[1] Yuan Li,[1] Tianqi Cai,[1,†] and Yicong Zheng[1]

[1] *Tencent Quantum Laboratory, Tencent, Shenzhen, Guangdong 518057, China*
[2] *These authors contributed equally. Sainan Huai, Kunliang Bu and Xiu Gu*

CONTENTS

I. Theoretical model 1
A. 2-qubit JPM scheme 1
B. 4-qubit JPM scheme 2

II. Extended experimental data 4

III. Device parameters 4

IV. Calibration procedure for joint parity measurement 5
A. Fundamental calibration 5
B. Gate calibration 6
1. Single-qubit gate calibration 6
2. Two-qubit gate calibration 7
3. Readout calibration 7
C. Joint calibration flow for the JPM scheme 7

V. Error Analyses 8
A. Error sources in the transversal parity measurement circuit 8
1. Decoherence 8
2. Calibration imperfection 8
B. Error sources in the longitudinal parity measurement circuit 8
1. Crosstalk 8
2. Leakage 10

VI. References 11

I. THEORETICAL MODEL

In this section, we review the theoretical model in the main text and give a derivation of the joint parity measurement (JPM) scheme via stimulated emission in detail. We will first derive the protocol for the weight-2 parity measurement scheme in the superconducting quantum system. Then, the weight-4 parity measurement scheme based on the sequential JPM unitary gates is further explored in the surface code architecture. Notice that two types of entangling gates (iSWAP gate and CZ gate) are verified to be available for experimental realization.

A. 2-qubit JPM scheme

We consider a generic superconducting qubit system of two data qubits (Q_1 and Q_2) connected to a syndrome qubit (Q_0) with the effective coupling strength g_i ($i = 1, 2$). The full system Hamiltonian under the rotating wave approximation is expressed as ($\hbar = 1$)

$$H = \sum_{i=0,1,2} \left(\omega_i a_i^\dagger a_i + \frac{\alpha_i}{2} a_i^\dagger a_i^\dagger a_i a_i\right) + g_1\left(a_1^\dagger a_0 + a_1 a_0^\dagger\right) + g_2\left(a_2^\dagger a_0 + a_2 a_0^\dagger\right), \tag{S1}$$

where ω_i and α_i are the bare frequencies and anharmonicities of the qubits Q_i ($i = 0 \sim 2$); a_i, $a_i^\dagger$ ($i = 0 \sim 2$) are the corresponding annihilation and creation operators.

We now explore the implementation of 2-qubit JPM scheme inspired by stimulated emission. To construct the collective coupling between the data qubits and syndrome qubit, the frequencies of the two data qubits are tuned into resonance and the coupling strength is set to be the same, i.e., $\omega_1 = \omega_2 = \omega_q$ and $g_1 = g_2 = g$. Then the Hamiltonian can be described as

$$H = \omega_0 a_0^\dagger a_0 + \omega_q J_z + g\left(aJ_+ + a^\dagger J_-\right) + \frac{\alpha_0}{2} a^\dagger a^\dagger a a. \tag{S2}$$

Here, we introduce the collective operators $J_+ = \sigma_1^{10} + \sigma_2^{10}$, $J_z = \sigma_1^z + \sigma_2^z$, where $\sigma_i^{n,m} = |n\rangle_i \langle m|$ is the $|m\rangle \to |n\rangle$ transition matrix element for qubit i. The corresponding Dicke state basis can be described as $\left|D^0\right\rangle = |00\rangle$, $\left|D^1\right\rangle = (|01\rangle + |10\rangle)/\sqrt{2}$, $\left|D^2\right\rangle = |11\rangle$ and

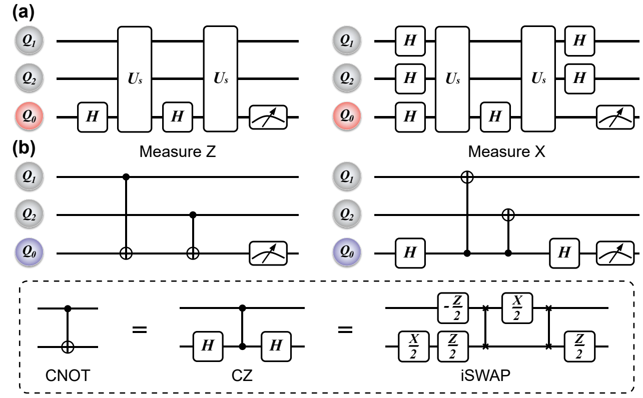

FIG. S1. **2-qubit parity measurement circuit comparison.** Parity measurement circuit for (a) 2-qubit JPM scheme and (b) 2-qubit CNOT scheme. Left panel: measure-Z detection circuit. Right panel: measure-X detection circuit. Specifically, the complete composition of the CNOT gate is depicted based on the superconducting qubit system.

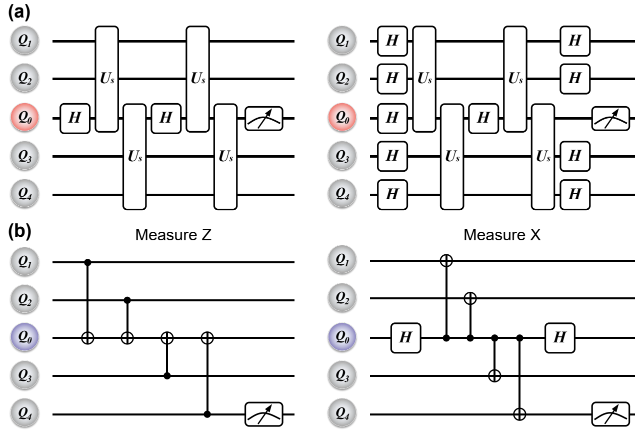

FIG. S2. **4-qubit parity measurement circuit comparison.** Parity measurement circuit for (a) 4-qubit JPM scheme and (b) 4-qubit CNOT scheme. Left panel: measure-Z detection circuit. Right panel: measure-X detection circuit.

$\left|d^1\right\rangle = (|01\rangle - |10\rangle)/\sqrt{2}$. In the following analysis, we denote the n-th energy level of the syndrome qubit as $|n\rangle$, and the collective basis of data qubits with k excitations as $\left|\psi^k\right\rangle$ ($\psi = D, d$). The syndrome-data qubit coupling induces transition from $|n\rangle\left|\psi^k\right\rangle$ as

$$\begin{aligned} a^\dagger J_- |n\rangle\left|\psi^k\right\rangle &= \sqrt{n+1} g g_-^k |n+1\rangle\left|\psi^{k-1}\right\rangle, \\ a J_+ |n\rangle\left|\psi^k\right\rangle &= \sqrt{n} g g_+^k |n-1\rangle\left|\psi^{k+1}\right\rangle. \end{aligned} \tag{S3}$$

The energy spacing between state $|n+1\rangle$ and $|n\rangle$ of the syndrome qubit is near resonant with the data qubit frequency, while other states are far off-resonant under the condition $\alpha_c \gg g$. We use δ to denote the small detuning ($\delta \lesssim g$) between $|n\rangle\left|\psi^k\right\rangle$ and $|n+1\rangle\left|\psi^{k-1}\right\rangle$. We find that even $|n\rangle$ is now fixed due to the anharmonicity of the syndrome qubit, $\left|\psi^k\right\rangle$ can still climb the eigenenergy ladder, and the detuning between $|n\rangle\left|\psi^{k+1}\right\rangle$ and $|n+1\rangle\left|\psi^k\right\rangle$ is also δ. Therefore the evolution of the system can be described by the doublet $|n\rangle\left|\psi^{k+1}\right\rangle \longleftrightarrow |n+1\rangle\left|\psi^k\right\rangle$ $\left(|n\rangle\left|\psi^k\right\rangle \longleftrightarrow |n+1\rangle\left|\psi^{k-1}\right\rangle\right)$, which follows the well-known two-level dynamics:

$$U_s = \cos\left(g'_\pm t\right) - i\sin\left(g'_\pm t\right) \boldsymbol{n}^\pm \cdot \tilde{\boldsymbol{\sigma}}_{\boldsymbol{n}}{}^\pm. \tag{S4}$$

Here, an overall phase $\exp(-i\delta t)$ is omitted, and $g'_\pm = \sqrt{(g g_\pm^k)^2 + \delta'^2}$ is the effective Rabi frequency, $\delta' = \delta/(2\sqrt{n+1})$ is the renormalized detuning. The vector $\boldsymbol{n}^\pm = \left(g g_\pm^k, 0, -\delta'/2\right)/g'_\pm$, and $\tilde{\boldsymbol{\sigma}}^\pm$ is the Pauli matrices vector acting on the states $|n\rangle\left|\psi^{k+1}\right\rangle \longleftrightarrow |n+1\rangle\left|\psi^k\right\rangle$ $\left(|n\rangle\left|\psi^k\right\rangle \longleftrightarrow |n+1\rangle\left|\psi^{k-1}\right\rangle\right)$.

Let us consider the evolution of state $|n\rangle\left|\psi^k\right\rangle$ generated by the circuit $H_0 U_s H_0$, i.e.,

$$H_0 U_s H_0 |n\rangle\left|\psi^k\right\rangle = \left(|n+1\rangle|\Psi_+\rangle + |n\rangle|\Psi-\rangle\right)/2. \tag{S5}$$

When the time t satisfies the condition $\sin(g'_+ t) = 0$, we have

$$|\Psi\rangle_\pm = (\cos(g'_- t) \mp \cos(g'_+))\left|\psi^k\right\rangle \tag{S6}$$

Thus the resulting probabilities of finding the syndrome qubit in state $|n+1\rangle$ (P_+^k) and state $|n\rangle$ (P_-^k) are

$$P_\pm^k = (1 \mp \cos(g'_- t)\cos(g'_+ t))/2, \tag{S7}$$

Besides, in order to modify the minus phase that may acquire during the above circuit (as shown in Fig. S1), another U_s should be applied to ensure the input state remaining constant.

When we consider the case of $\omega_q = \omega_0$ (iSWAP-case) or $\omega_q = \omega_0 + \alpha$ (CZ-case), the effective Hamiltonian in the interaction picture with the two-level approximation can then be expressed as Eq.(1) in the main text.

B. 4-qubit JPM scheme

We turn to a generalization of the JPM scheme to include four data qubits to realize weight-4 parity measurement in the surface code architecture. The data qubits are chosen as $Q_1 \sim Q_4$ and each one is connected to the syndrome qubit (Q_0) with the effective coupling strength g_i ($i = 1 \sim 4$). The full system Hamiltonian is similarly written as

$$\begin{aligned} H = &\sum_{i=0\sim 4}\left(\omega_i a_i^\dagger a_i + \frac{\alpha_i}{2} a_i^\dagger a_i^\dagger a_i a_i\right) \\ &+ g_1\left(a_1^\dagger a_0 + a_1 a_0^\dagger\right) + g_2\left(a_2^\dagger a_0 + a_2 a_0^\dagger\right) \\ &+ g_3\left(a_3^\dagger a_0 + a_3 a_0^\dagger\right) + g_4\left(a_4^\dagger a_0 + a_4 a_0^\dagger\right). \end{aligned} \tag{S8}$$

Notice that the rotating wave approximation is implemented in this equation.

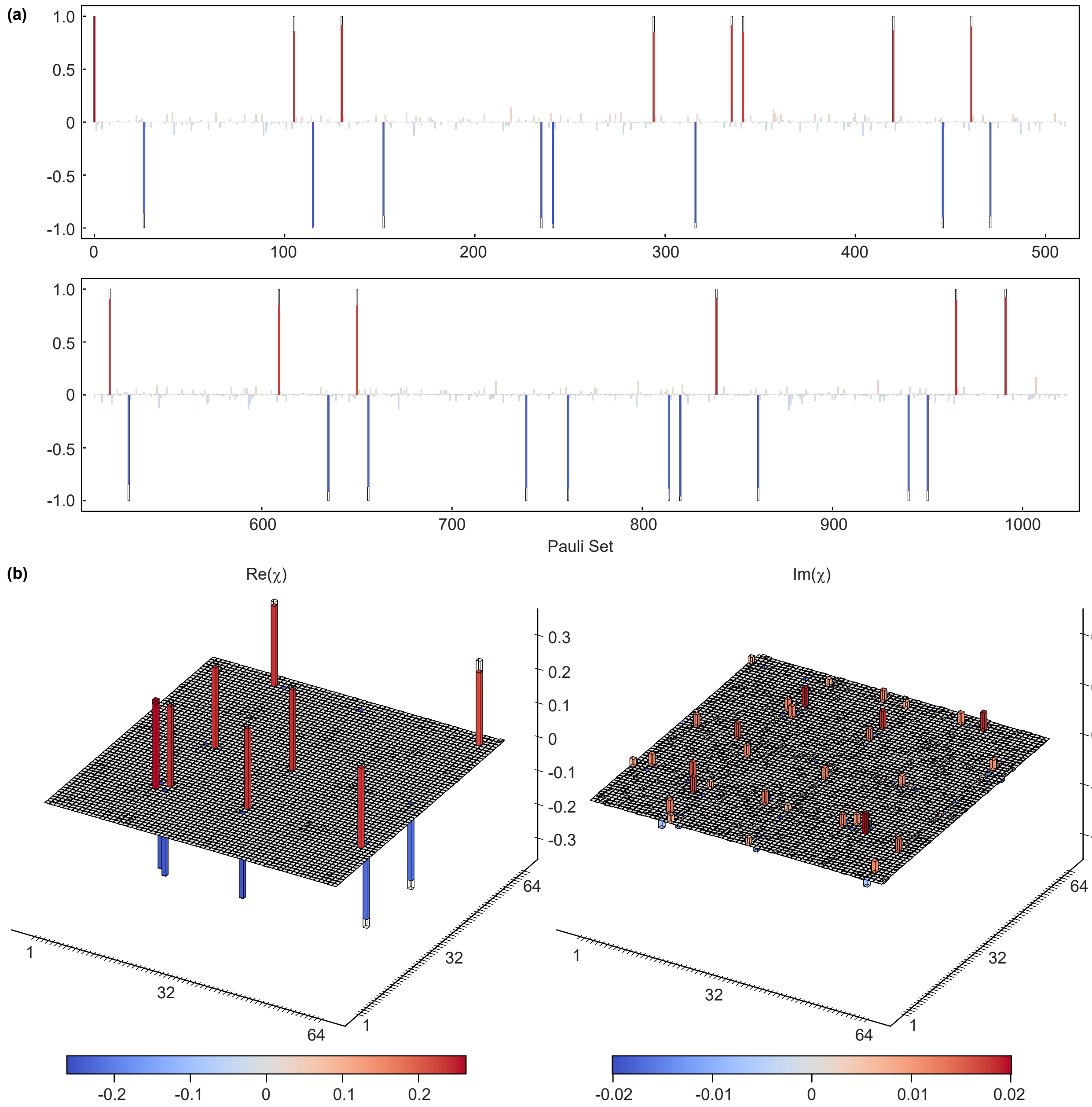

FIG. S3. **Calibration of the 4-qubit iSWAP-based JPM scheme.** (a) The expectation of the Pauli set measured in 4-qubit JPM scheme with the initial states $|+++++\rangle$ ($|Q_2Q_1Q_0Q_4Q_3\rangle$). The numbers on the horizontal axis represent the order of the Pauli set in terms of I, X, Y, Z for each qubit. (b) Phase accumulation during the 4-qubit JPM unitary gate is extracted via the quantum state tomography (QST) with the initial states $|+++++\rangle$.

Obviously, the simplist and most direct idea is to set $\omega_i = \omega_0$ or $\omega_i = \omega_0 + \alpha_0$ ($i = 1 \sim 4$) for utilizing the 4-qubit collective iSWAP gates or CZ gates between these data qubits and the syndrome qubit. However, the derivation reveals that we can't find a time t to align all the even (odd) parity oscillations, and thus the output state will be changed after passing through the parity measurement circuit which is unacceptable in the surface code. Therefore, we here take a different route by introducing the sequential JPM unitary gates.

The sequential JPM unitary gate is achieved by dividing the gate procedure into two segments. Taking iSWAP-based JPM scheme as an example, in the first part ($t = 0 \sim t_{seg}$), the frequencies of two data qubits (Q_1 and Q_2 as an example) and syndrome qubit (Q_0) are set to be resonant, i.e. $\omega_0 = \omega_1 = \omega_2$. Then the first segment Hamiltonian is described as

$$H_{seg} = g_{seg_1}\sigma_0^{10}\left(\sigma_1^{01} + \sigma_2^{01}\right) + H.c, \, (0 \le t < t_{seg}) \quad \text{(S9)}$$

where we set $g_1 = g_2 = g_{seg_1}$. Next, in the second seg-

ment ($t = t_{seg} \sim t_{gate}$), the frequencies of the remaining two data qubits (Q_3 and Q_4) are set to be resonant with the syndrome qubit (Q_0), i.e. $\omega_0 = \omega_3 = \omega_4$ with the Hamiltonian written as ($g_3 = g_4 = g_{seg_2}$)

$$H_{seg} = g_{seg_2}\sigma_0^{10}\left(\sigma_3^{01} + \sigma_4^{01}\right) + H.c.\,(t_{seg} \le t \le t_{gate}) \tag{S10}$$

The total Hamiltonian is the composition of the two parts as $H = H_{seg}(t)$ and the unitary evolution is then expressed as $U = e^{-iHt} = U_2U_1$ with $U_k = e^{-iH_{seg_k}t}$ ($k = 1, 2$). To construct the parity measurement circuit in the surface code, we continue to utilize the similar circuit as shown in Fig. S2. The theoretical result clearly demonstrates that when the input state of data qubits ($Q_1 \sim Q_4$) are in an even number of excitation, the state of syndrome qubit (Q_0) remains unchanged. Otherwise, the state of syndrome qubit is flipped if the input state of data qubits are in an odd excitation number. In practice, the situation of sequential CZ-based JPM gate is similar except that all the single-qubit gates are extended to the subspace spanned by the first excited state and the second excited state.

II. EXTENDED EXPERIMENTAL DATA

In our experiment, we demonstrate the weight-2 (weight-4) parity measurement with the (sequential) JPM unitary gates in the 2-qubit (4-qubit) JPM scheme. Here we further present the extended data during the experiment in detail, as well as the results compared with the simulations to more clearly validate the JPM process. At the same time, we will also show relevant experimental data of the parity measurement under CNOT scheme as a supplement.

In the main text, we have discussed the experimental demonstration of the unitary gate for the JPM scheme which critically decide the fidelity of the parity measurement. Figure 3 in the main text mainly shows the expectation of the Pauli set and the QST results when calibrate the 2-qubit iSWAP-based JPM scheme. Here we further give the corresponding experimental results when we calibrate the unitary gate in the 4-qubit JPM scheme as a whole with sequential JPM unitary gates, as depicted in Fig. S3. Given the initial state $|+++++\rangle$ ($|Q_2Q_1Q_0Q_4Q_3\rangle$), the QST measurement of the final state after the 4-qubit JPM unitary gate can help to extract the phase accumulation and check the gate calibration procedure. The detailed calibration flow for both 2-qubit JPM scheme and 4-qubit JPM scheme can be found in Sec. IV.

The CNOT scheme for realizing the parity measurement primarily depends on the single-qubit gates and native two-qubit entangling gates [1]. In our superconducting qubit system, CZ gates are utilized as the two-qubit gates to perform CNOT gate. Therefore, the calibration and the gate fidelity of the CZ gates directly decide the parity detection fidelity. Figure S4 exhibits the results of the CZ gates which have been used in the experiment. The detailed calibration flow for single-qubit gates and CZ gates can be found in Sec. IV and the corresponding gate fidelity is recorded in Table S1.

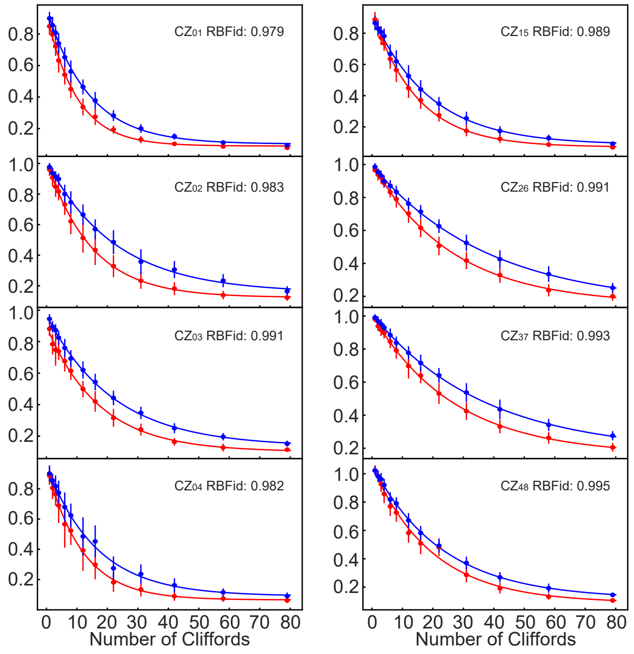

FIG. S4. **Randomized Benchmarking (RB) of CZ gates.** The CZ gate fidelity is acquired according to the reference RB and interleaved RB between each adjacent qubit pairs in our superconducting quantum processor.

III. DEVICE PARAMETERS

Our device is mounted in an aluminium sample holder at a base temperature of ~ 10 mK in the dilution refrigerator as mentioned in the Methods section in the main text. The experimental setup are depicted in Fig. S5. Meanwhile, the fundamental information of the device is listed in Table S1. We monitor T_1, T_2 and $T_{2,echo}$ for several days and the recorded data are the average results. The coupler frequency is measured by taking advantage of the dispersive shift between qubit and coupler. The qubit-coupler coupling strength $g_{c,L}$, $g_{c,R}$ is measured through the chevron pattern generated from the SWAP procedure. Meanwhile, the qubit-qubit direct coupling strength $g_{c,d}$ is fitted via measuring the qubit-qubit effective coupling strength. To confirm the coupler frequency, a long π pulse is executed to the measure qubit with the rectangular pulse of different frequency applying to the coupler. When the coupler is excited, the measure qubit will not be able to maintain in the $|1\rangle$

TABLE S1. Device parameters. ω_r is the cavity frequencies for readout. ω_j, α_j (ω_c, α_c) are the idle frequencies and anharmonicities for each qubit (coupler). The coupler idle frequencies chosen here are required to maximally suppress the ZZ crosstalk between adjacent qubits. T_1, T_2 are the corresponding energy relaxation time and Ramsey dephasing time. The average readout fidelities based on error mitigation are labeled by $F_{avr,j}$ where the outcomes are reconstructed through the Bayes' rule. $g_{c,L}$, $g_{c,R}$ are the qubit-coupler coupling strength measured at the qubit frequency and $g_{c,d}$ is the qubit-qubit direct coupling strength fitted via measuring the qubit-qubit effective coupling strength. Single-qubit (two-qubit) gate errors e_{sq} ($e_{tq,CZ}$) are measured with Randomized Benchmarking (RB). Notice that the data was obtained during the experiment.

Single-qubit gates

	Q_0	Q_1	Q_2	Q_3	Q_4	Q_5	Q_6	Q_7	Q_8
ω_r (GHz)	6.874	6.825	6.931	6.901	6.845	6.786	6.991	6.961	6.806
ω_j (GHz)	3.988	4.187	4.464	4.668	4.404	4.359	4.641	4.498	4.223
$\alpha_j/2\pi$ (MHz)	−260	−258	−255	−250	−254	−258	−253	−257	−264
T_1 (μs)	35.3	31.6	29.5	27.7	33.9	34.3	33.3	22.1	31.8
T_2 (μs)	11.0	10.2	32.6	38.2	9.1	5.6	43.1	24.1	4.3
$F_{avr,j}$ (%)	97.0	95.9	95.6	96.6	96.6	96.5	94.5	94.8	92.5
e_{sq} (%)	0.19	0.27	0.09	0.09	0.14	0.06	0.07	0.07	0.06

Two-qubit gates

	C_{01}	C_{02}	C_{03}	C_{04}	C_{15}	C_{26}	C_{37}	C_{48}
ω_c (GHz)	5.020	5.445	5.570	5.335	5.325	5.595	5.695	5.355
$\alpha_c/2\pi$ (MHz)				~ -140				
$g_{c,L}$ ($g_{c,R}$)$/2\pi$ (MHz)				~ 62				
$g_{c,d}/2\pi$ (MHz)				~ 4.1				
$e_{tq,CZ}$ (%)	2.1	1.7	0.9	1.8	1.1	0.9	0.7	0.5

state and then the population will changed in the spectrum [2]. The static ZZ crosstalk can also be measured with the Ramsey-type experiment. The acquired residual ZZ can be calculated as $\xi_{ZZ} = E_{11} - E_{10} - E_{01} + E_{00}$

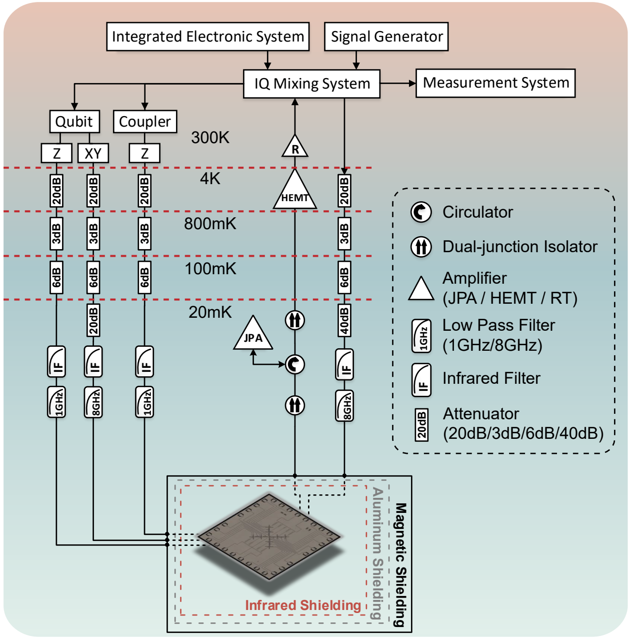

FIG. S5. **Measurement setup.** The gradient color in the background represents the temperature layer of the dilution refrigerator from room temperature to the lowest temperature.

where E_{00}, E_{01}, E_{10}, E_{11} are eigenenergy for the hybrid qubit system. In practice, we select the coupler frequencies to minimize the ZZ crosstalk between each adjacent qubit pairs. Note that here the frequency detuning between certain qubit pairs like Q_0 and Q_2 has exceeded the anharmonicity of the qubit, therefore theoretically there will be no absolutely zero point for the static ZZ crosstalk [3]. However, simulations reveal that we can still acquire a relatively low static ZZ crosstalk when the coupler frequency is suitably adjusted, as shown in Table S1. Calibration of single-qubit gates, two-qubit gates and readout can be found in Sec. IV.

IV. CALIBRATION PROCEDURE FOR JOINT PARITY MEASUREMENT

The calibration process is critical for both the JPM scheme and the CNOT scheme as the gate operations and readout will influence the parity detection fidelity. We now give the calibration flow in detail.

A. Fundamental calibration

In this part, we setup the idle condition for all the qubits and couplers, acquire the basic parameters for the experiment, optimize the hardware-based electronic system and prepare the fundamental gate information for the following gate calibration. We list the fundamental calibration flow as followed

- Roughly shift the qubit to the sweet spot via the readout cavity response as a function of the qubit Z bias. Perform the power Rabi oscillation to find the initial π pulse for the qubit. Perform Ramsey measurement to calibrate the frequency.

- Perform qubit spectroscopy as a function of the qubit Z bias to acquire the relation between qubit frequency and the bias.

- Perform qubit-coupler SWAP procedure to determine the qubit-coupler coupling strength $g_{c,L}$ (or $g_{c,R}$).

- Roughly determine the coupler frequencies via scanning the anti-crossing between couplers and the adjacent qubits based on performing qubit spectroscopy. Adjust the coupler frequency to the approximate position based on the simulations where static ZZ crosstalk is relatively slow. Perform Ramsey-type experiment to precisely measure the coupler's frequency with the help of the dispersive shift between qubit and coupler.

- Perform qubit-qubit SWAP procedure as a function of the coupler Z bias. Fit the corresponding effective coupling strength J with the coupler frequency to acquire the qubit-qubit direct coupling strength $g_{c,d}$.

- Calibrate LO leakage and the sideband leakage resulting from the IQ mixers.

- Scanning qubit decoherence time T_1 as a function of the qubit Z bias to locate the potential problematic frequencies attributed to the TLS defects, stray couplings, leakage channels and so on.

- Calibrate the timing between the qubit microwave pulse and the flux bias pulse, the qubit flux pulse and the coupler flux pulse.

B. Gate calibration

Now we turn to the calibration of the gate which is essential for the parity detection. We divide this section into three parts including single-qubit gate calibration, two-qubit gate calibration and readout calibration.

1. *Single-qubit gate calibration*

In the parity detection, the commonly used single-qubit gate is Hadamard gate which can be transformed into $R_y(\frac{\pi}{2})$ in many situations. Therefore, delicate single-qubit calibration is critical to the parity detection fidelity. We here demonstrate our calibration procedure as

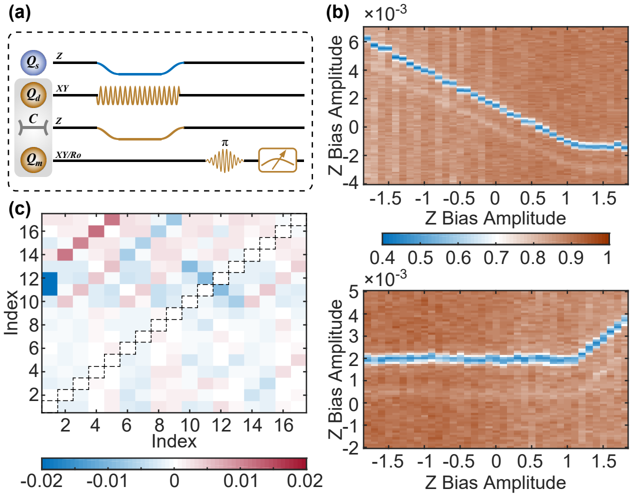

FIG. S6. **Calibration of Z crosstalk.** (a) Pulse sequence for measuring the Z crosstalk response of target coupler to the source qubit or coupler. Here, the target coupler Z line is drived (measured) with Q_d (Q_m). (b) The spectrum of the uncorrected (upper panel) and corrected (bottom panel) qubit-to-coupler Z crosstalk. (c) The Z crosstalk matrix obtained via measuring the response in (b), with the index listed in order from qubits $Q_0 - Q_8$ to the couplers $C_{01} - C_{48}$.

- Finding the appropriate idle frequencies ω_j for all the computational qubits with consideration of the frequency distribution to avoid frequency crowding, potential problematic frequency positions and satisfy gate operation requirements.

- Measure the static ZZ crosstalk as a function of the coupler frequency to seek for the idle frequency ω_c for all the couplers where the ZZ crosstalk between the adjacent qubits are minimized.

- Calibrate the Z crosstalk of both qubits and couplers with scanning the complete Z response from qubit-qubit and qubit-coupler. Z-crosstalk calibration matrix is then acquired as found in Fig. S6 [4].

- Measure T_1, T_2, $T_{2,echo}$ at the idle frequencies for all the qubits to allocate the basic properties.

- Perform single-qubit microwave pulse calibration delicately containing power, gate time, leakage suppression with DRAG [5] and waveform optimization. Ensure the single-qubit gate fidelity with AllXY [6] and further optimize the gate fidelity with isolated Randomized Benchmarking (RB).

- Perform simultaneous Randomized Benchmarking to finally check out the potential error sources like residual ZZ crosstalk and microwave crosstalk. Slightly adjust the qubit frequencies and the pulse parameters based on the simultaneous RB.

2. Two-qubit gate calibration

In the CNOT scheme, CNOT gate can be realized with iSWAP gate or CZ gate. Here, for comparison with the JPM scheme, we select the controlled-Z (CZ) gate as the basic two-qubit entangling gate. CZ gate is commonly used in superconducting transmon system as it contains several advantages like fast gate time, high fidelity and convenient phase calibration. Similarly, we follow the calibration procedure as

- Calibrate the qubit and coupler Z pulse distortion to suppress the influence on the two-qubit gates.
- Scan the oscillation between $|101\rangle$ and $|200\rangle$ (or $|002\rangle$) (in the order of $|\text{qubit, coupler, qubit}\rangle$) to roughly ensure the oscillation frequency and the gate time for two qubits.
- Scan the oscillation between $|101\rangle$ and $|020\rangle$ to check out the threshold of leakage from qubits to coupler. Determine the operation frequencies of the couplers when performing the CZ gates.
- Perform phase calibration with the Ramsey experiment. Determine the frequencies of the qubits and the accurate gate time with the requirement that the phase accumulation of the qubit-qubit $|11\rangle$ state should be π.
- Execute the quantum process tomography (QPT) or the isolated RB to measure the gate fidelity. Further improve the gate fidelity based on the pulse optimization with the help of RB.
- Execute the simultaneous RB to check out the potential residual error sources in the circuit and slightly adjust the gate parameters for multiqubit operations.

3. Readout calibration

To maximum the readout fidelity and suppress the error from the readout procedure, delicate calibration should be adopted in the dispersive readout process. We here list the calibration flow for the readout as

- Measure the readout delay time based on the electronic system to eliminate potential delay error.
- Perform the cavity measurement of S parameter in the function of the readout power, readout time and readout frequency. Determine the most appropriate readout parameters based on readout fidelity.
- Measure the single-qubit readout fidelity continuously to acquire the average readout calibration matrix for each qubit which is used to reconstruct the qubit readout results based on the Bayes' rule.
- Calibrate the multiqubit readout measurement with the simultaneous readout calibration to suppress the influence from the readout crosstalk.

C. Joint calibration flow for the JPM scheme

To perform the JPM scheme, several calibrations should be carefully implemented to avoid unwanted gate error. Here, we take 2-qubit iSWAP-based JPM scheme as an example to list the calibration flow and the similar procedure can be executed to calibrate 4-qubit JPM scheme (Suppose Q_1, Q_2 are data qubits and Q_0 is syndrome qubit with couplers C_{01}, C_{02}).

- Using the standard two-qubit iSWAP gate procedure and depending on coupling strength requirements, prioritize the approximate frequency for couplers C_{01}, C_{02} to be tuned when implementing the 2-qubit JPM scheme.
- The same iSWAP gate procedure is separately repeated for each two-qubit Q_0, Q_1 and Q_0, Q_2 to calibrate the resonance positions of all qubits in the implementation of the JPM scheme. It should be noted that this time the two couplers C_{01}, C_{02} need to be adjusted together in the implementation of the iSWAP gate. This is to maximally restore the pulse situation in the JPM scheme for calibration so as to reduce the effect of the dispersive shift due to the always-on coupling between qubits and couplers.
- Measure the population oscillations with the $|010\rangle$ ($|Q_1Q_0Q_2\rangle$) initial state in the JPM scheme, as depicted in Fig. 2(d) in the main text.
- Fine tune the qubit frequencies and coupler frequencies for the JPM scheme with the measurement of periodic population oscillation based on the simulation results and the coherence lifetime of qubits. The unitary gate time for the 2-qubit JPM gate can then be extracted when the state $|010\rangle$ returning to the initial.
- Measure the phase accumulation for each qubits in the JPM scheme via the QST with the preparation of $|+++\rangle$ initial state. Moreover, the QST process is reused to further carefully improve the unitary gate fidelity of the JPM scheme with adjusting qubit frequencies, coupler frequencies and gate time.
- Measure the QPT for the 2-qubit JPM scheme to finally check the calibration procedure.

V. ERROR ANALYSES

In this section, we further carry out the error analyses for the JPM scheme (especially the collective-iSWAP based JPM scheme) in detail, exploring the error sources in the transversal and longitudinal parity measurement circuit based on the surface code architecture.

A. Error sources in the transversal parity measurement circuit

1. *Decoherence*

Generally, the major error existed in the transversal parity measurement circuit may source from the multi-qubit entangling gates. For the JPM scheme, this refers to the JPM unitary gate U_s which can be viewed as a three-qubit entangling gate [7]. In the experiment, the primal error that the JPM unitary gate suffers from is the qubit decoherence $T_1^{Q_i}$, $T_\phi^{Q_i}$ ($i = 0, 2, 4$). To quantify the infidelity contribution of the energy relaxation and pure dephasing mechanism, we first use the theoretical formula [8] $\Delta_{F_{dec}} = \sum_{i=0,2,4} \frac{t_{gate}}{2T_1^{Q_i}} + \frac{t_{gate}}{2T_\phi^{Q_i}}$ and arrive at the evaluation result $\Delta_{F_{dec,cal}} = 1.1\%$. Furthermore, we numerically simulate the gate procedure with the consideration of the qubit decoherence for verifying its effects on gate infidelity, as revealed in Fig. S7(a). Ignoring the pure dephasing and setting the energy relaxation time of Q_4 to be infinite, we can plot the QPT gate fidelity of the JPM unitary gate varying with the $T_1^{Q_0}$ and $T_1^{Q_2}$ (the similar result happens for selecting any two of the three qubits). The exact induced gate infidelity from the qubit decoherence is finally simulated with the experimental parameters to be around $\Delta_{F_{dec,sim}} = 1.0\%$ which is basically consistent with the theoretical calculation result.

2. *Calibration imperfection*

Another potential error that may be brought into the JPM unitary gate is the incomplete calibration. As we mentioned in Experimental demonstration in the main text, the robust JPM unitary gate depends on two basic requirements, i.e., the fully resonant frequency and equal coupling strength. For instance, Fig. S7(b) explores the effect from the mismatch of the coupling strength in the simulation. It can be clearly seen that the growth of the error follows a parabola shape and the coupling deviation leads to nonnegligible effect on gate infidelity. Similarly, the frequency mismatch may also cause extra error. Therefore, the whole calibration of the JPM unitary gate should be carefully treated and optimized.

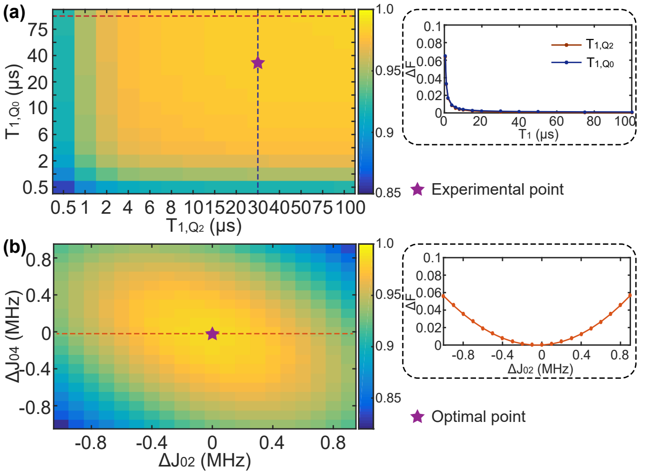

FIG. S7. **Simulations for error sources in the transversal parity measurement circuit.** (a) Error from the qubit decoherence. (b) Error from the coupling mismatch. The simulations are carried out with the Qutip package in Python [9] (see Methods in the main text). To simulate the influence of the qubit energy relaxation time, the Lindblad operator is chosen as a_i ($i = 0, 2, 4$). Similarly, the Lindblad operator is chosen as $a_i^\dagger a_i$ ($i = 0, 2, 4$) if we tend to simulate the pure dephasing effects. The right panel inside the dotted box is an enlarged view of the corresponding dotted line in the left panel.

B. Error sources in the longitudinal parity measurement circuit

1. *Crosstalk*

Typically, the crosstalk can be divided into classical crosstalk (e.g. Z crosstalk and microwave X crosstalk) and quantum crosstalk (e.g. ZZ crosstalk). For classical crosstalk, the Z crosstalk mainly arises from the uneven potential distribution on the chip. As mentioned in Sec. IV, we have employed a Z crosstalk matrix method for precise evaluation and mitigation of the crosstalk as shown in Fig. S6. Meanwhile, the microwave X crosstalk may cause leakage out of the computational subspace, e.g., parasitic $|1\rangle \to |2\rangle$ leakage, when the driving frequency of a source qubit is close to the f_{12} of a receiver qubit. This situation primarily occurs during single-qubit gate operations with all the qubits positioned at idle frequencies, and optimization methods such as utilizing compensation pulse to the receiver qubit can effectively mitigate the leakage [10]. In practice, there are no distinctions on these classical crosstalk for the JPM scheme and the CNOT scheme. However, differences may arise for these two schemes when considering the quantum crosstalk, especially ZZ crosstalk induced from the anharmonicity of transmon qubits. Here, we focus on the following three types.

Static ZZ crosstalk. The first type of ZZ crosstalk

occurs during the execution of single-qubit gates, commonly recognized as the static ZZ crosstalk and also made no distinction for the JPM scheme and the CNOT scheme. Taking parity measurement circuit based on surface code as an example, the static ZZ crosstalk may generate when the ZZ interaction between neighbouring qubits are not effectively turned off, marked as the red dotted line in Fig. S8(a). In order to mitigate such crosstalk error, the tunable couplers between adjacent qubits should be correctly biased to the appropriate frequencies [2].

ZZ crosstalk from spectator qubits. The second type of ZZ crosstalk happens during the execution of multiqubit entangling gates. In the CNOT scheme, global parallel CZ gates appear in a specific order and direction, while in the JPM scheme, global parallel multiqubit JPM unitary gates (collective iSWAP-based or collective CZ-based) are also required. We now investigate the impact of ZZ crosstalk from spectator qubits surrounding these multiqubit entangling gates. In the case of one CZ gate in the CNOT scheme as shown in Fig. S8(b), six pairs of ZZ interactions may affect the two gate qubits; whereas for one JPM unitary gate in the JPM scheme as shown in Fig. S8(c), only four pairs of ZZ interactions are influential (with some spectator qubits remaining free and not participating in multiqubit operations, thus their influence can be disregarded). However, due to limitations imposed by frequency crowding during gate operations, effectively eliminating ZZ crosstalk between gate qubits and spectator qubits becomes challenging. Here we further validate the effect of ZZ crosstalk during gate operations through simulation. As depicted in Fig. S8(d), it can be observed that an increase in number of spectator qubits (resulting in an increased ZZ crosstalk) exacerbates infidelity of the gates. Moreover, we should further verify the fidelity tolerance of both the JPM gate and the CZ gate towards single ZZ interaction, which we define as $FT = F(\xi_{ZZ} = \xi) - F(\xi_{ZZ=0})$ (ξ_{ZZ} represents the ZZ interaction between one gate qubit and one spectator qubit). This is because during gate operations, some gate qubits and spectator qubits exhibit a frequency difference within the range of couplers capable of suppressing ZZ crosstalk. Under such circumstance, investigating the fidelity tolerance of single ZZ interaction with respect to gate operations will showcase the robustness of the gate operation itself against correlated errors. We conduct simulations to validate this aspect as illustrated in Fig. S8(e). Notably, in the CNOT scheme, when performing CZ gates, two qubits are distributed at two frequencies, potentially leading to increased frequency crowding globally; whereas in collective iSWAP-based JPM scheme, three qubits are distributed resonantly and share a common frequency position. Consequently, during parallel gates execution, there will be less frequency crowding issues compared to the CNOT scheme which facilitates easier elimination of ZZ errors.

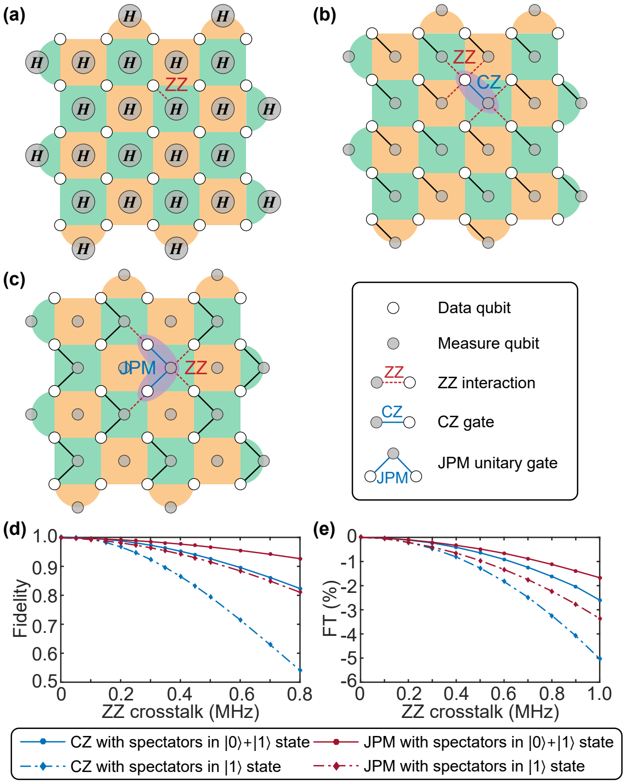

FIG. S8. **ZZ crosstalk in the surface code architecture.** (a) Static ZZ crosstalk during single-qubit gate operations. The red dotted line represents the potential static ZZ crosstalk may occur when the frequency of coupler is inappropriate. (b) ZZ crosstalk from spectator qubits may cause correlated error when CZ gates are simultaneously implemented during the parity measurement of surface code. Here up to six adjacent spectator qubits may generate six pairs of ZZ error to gate qubits circled by purple shade. (c) ZZ crosstalk from spectator qubits may cause correlated error when JPM unitary gates are simultaneously implemented during the parity measurement of surface code. Here only up to four adjacent spectator qubits may generate four pairs of ZZ error to gate qubits circled by purple shade (notice that although gate qubits are surrounded by seven spectator qubits, three of them are free and can be biased far away in principle). (d) Fidelity vs. ZZ crosstalk for both the JPM gate and the CZ gate. The effective spectator qubits in (b) and (c) are all initialized in $|1\rangle$ states or $|0\rangle + |1\rangle$ states. (e) Fidelity tolerance (FT) vs. ZZ crosstalk with only one spectator qubit for both the JPM gate and the CZ gate. The spectator qubit is initialized in $|1\rangle$ states or $|0\rangle + |1\rangle$ states.

Dynamic ZZ crosstalk. The third type of ZZ crosstalk is parasitic ZZ crosstalk during gate procedure (or defined as dynamic ZZ crosstalk [11]). CZ gate and collective CZ-based JPM gate do not have parasitic ZZ crosstalk, but may exist leakage between $|01\rangle$ and $|10\rangle$ of two gate qubits [12], while iSWAP-based JPM gate may encounter parasitic ZZ crosstalk. However, the ZZ-free iSWAP gate scheme may further provide a new idea to optimize JPM gate free of parasitic ZZ crosstalk [13].

2. Leakage

Leakage to coupler state. In order to achieve faster entangling gate, the couplers should be biased to a lower frequency positions during multiqubit gate operations. However this may potentially lead to energy exchange between qubits and couplers, resulting in leakage. Here we introduce leakage interference method based on the JPM scheme to balance the reduction of qubit-coupler leakage and the speed of multiqubit gate operation [2, 13].

As an example, we consider the implementation of the collective iSWAP-based JPM unitary gate utilized in our experiment where the most common leakage happens from qubits to couplers: Assuming that two data qubits Q_1, Q_2 and one syndrome qubit Q_0 are tuned into resonance, together with the corresponding two couplers C_1 (between Q_0 and Q_1), C_2 (between Q_0 and Q_2) adjusted to ensure the consistency of the effective coupling strength. If no leakage is happened during the gate operation, the population of quantum state $|10101\rangle$ (ordered as $|Q_1C_1Q_0C_2Q_2\rangle$) will not change over time. Once the leakage occurs from the qubits to couplers, the $|10101\rangle$ state may transformed to other potential leakage channels like $|01101\rangle$, $|00111\rangle$ and $|01110\rangle$, resulting in the variation of population evolution. Therefore, we can obtain the leakage interference image from this JPM unitary gate procedure with varied coupler frequencies via measuring the population evolution of $|10101\rangle$ state. To verify our proposed leakage interference protocol for JPM unitary gate, we simulate this procedure through QuTip package in Python [9] based on the experimental gate parameters with pulse level, as shown in Fig. S9(a). It is obvious that when the coupler frequencies are lower than the threshold, marked as the red dotted line, the population of $|10101\rangle$ manifests relatively large oscillating ripples, revealing the appearance of non-negligible leakage to other potential leakage channels.

Similarly, we can also propose a leakage interference protocol for CZ gate: Assuming that two qubits Q_1, Q_2, together with the coupler C, are initialized to $|001\rangle$ state (ordered as $|Q_1CQ_2\rangle$), followed by implementing CZ gate procedure with varied coupler frequency. If no leakage is happened during the gate operation, the population of quantum state $|001\rangle$ will not change over time. Once the leakage occurs from the qubit to coupler, the $|001\rangle$ state may appear oscillating ripples, as the simulation results shown in Fig. S9(b). We can easily extract the lower bound for coupler frequency, marked as the red dotted line, to be higher than that in JPM unitary gate. In practice, this is not surprising since the frequency of one qubit in CZ gate is tuned higher to satisfy the resonance between two qubits $|11\rangle$ and $|02\rangle$ (or $|20\rangle$) while qubits in the JPM unitary gate are all positioned with the same frequencies, see Sec. IV. Therefore, leakage in CZ gate seems to be easier to happen compared with the JPM unitary gate, thereby the threshold of coupler frequencies for collective iSWAP-based JPM unitary gate is lower.

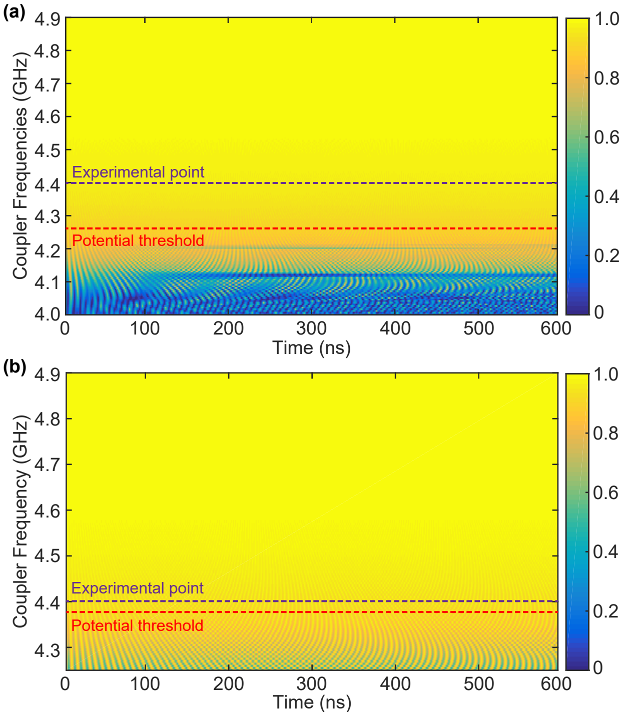

FIG. S9. **Leakage interference.** Simulation results of leakage interference for (a) collective iSWAP-based JPM unitary gate and (b) CZ gate based on the QuTip in Python. The system quantum states are initialized as (a) $|10101\rangle$ (ordered as $|Q_1C_1Q_0C_2Q_2\rangle$) and (b) $|001\rangle$ (ordered as $|Q_1CQ_2\rangle$)). Notice that the range of the vertical axis in the two images is different because the coupler cannot be tuned lower than qubits' frequencies when implementing gate operations. The purple dotted line represents the experimental frequencies for couplers while the red dotted line marked in the figure represents the potential lower bound (here we choose around 10% leakage as the threshold considering the balance between the time and fidelity of the quantum gate) for implementing gate operation. It can be easily found that the threshold in (a) is lower than that in (b), thereby the gate operation time for collective iSWAP-based JPM unitary gate can be further reduced.

Leakage to qubit higher excited state. Another leakage may exist from the qubit $|1\rangle$ state to $|2\rangle$ state during the execution of the surface code circuit. If we ignore such leakage in experiment, then after a certain circuit time, the $|2\rangle$ state occupation in the circuit will gradually rise, and eventually exceed the data space occupation [10]. Generally, the leakage of $|1\rangle$ to $|2\rangle$ may appear in heating, single-qubit gate operations (which can be suppressed via DRAG [5]) and multiqubit gate operations. For the JPM scheme and the CNOT scheme, the impact of leakage in heating and single-qubit gate operations is similar, and the major difference comes from the multiqubit gate operations. In the CNOT scheme, the diabatic CZ gate primarily employs swap process $|11\rangle \leftrightarrow |02\rangle$ (or $|20\rangle$)

to accumulate phases. Consequently, the occurrence of dephasing during this process may lead to leakage from $|11\rangle$ state to $|02\rangle$ (or $|20\rangle$) state. Conversely, in the JPM scheme, the collective iSWAP-based JPM gate only involves swap process $|10\rangle \leftrightarrow |01\rangle$ and thereby may not directly impact qubit higher excited states.

VI. REFERENCES

* guxiu1@gmail.com
† tianqicai@tencent.com

[1] M. Takita, A. D. Córcoles, E. Magesan, B. Abdo, M. Brink, A. Cross, J. M. Chow, and J. M. Gambetta, "Demonstration of weight-four parity measurements in the surface code architecture," Physical Review Letters **117**, 210505 (2016).

[2] X. Li, T. Cai, H. Yan, Z. Wang, X. Pan, Y. Ma, W. Cai, J. Han, Z. Hua, X. Han, *et al.*, "Tunable coupler for realizing a controlled-phase gate with dynamically decoupled regime in a superconducting circuit," Physical Review Applied **14**, 024070 (2020).

[3] Y. Xu, J. Chu, J. Yuan, J. Qiu, Y. Zhou, L. Zhang, X. Tan, Y. Yu, S. Liu, J. Li, *et al.*, "High-fidelity, high-scalability two-qubit gate scheme for superconducting qubits," Physical review letters **125**, 240503 (2020).

[4] H. Li, Y.-Y. Wang, Y.-H. Shi, K. Huang, X. Song, G.-H. Liang, Z.-Y. Mei, B. Zhou, H. Zhang, J.-C. Zhang, *et al.*, "Observation of critical phase transition in a generalized aubry-andré-harper model on a superconducting quantum processor with tunable couplers," arXiv e-prints , arXiv (2022).

[5] F. Motzoi, J. M. Gambetta, P. Rebentrost, and F. K. Wilhelm, "Simple pulses for elimination of leakage in weakly nonlinear qubits," Physical Review Letters **103**, 110501 (2009).

[6] M. Reed, *Entanglement and quantum error correction with superconducting qubits* (Lulu. com, 2013).

[7] C. W. Warren, J. Fernández-Pendás, S. Ahmed, T. Abad, A. Bengtsson, J. Biznárová, K. Debnath, X. Gu, C. Križan, A. Osman, *et al.*, "Extensive characterization of a family of efficient three-qubit gates at the coherence limit," arXiv preprint arXiv:2207.02938 (2022).

[8] A. N. Korotkov, "Error matrices in quantum process tomography," arXiv preprint arXiv:1309.6405 (2013).

[9] J. R. Johansson, P. D. Nation, and F. Nori, "Qutip: An open-source python framework for the dynamics of open quantum systems," Computer Physics Communications **183**, 1760 (2012).

[10] "Suppressing quantum errors by scaling a surface code logical qubit," Nature **614**, 676 (2023).

[11] X. Han, T. Cai, X. Li, Y. Wu, Y. Ma, Y. Ma, J. Wang, H. Zhang, Y. Song, and L. Duan, "Error analysis in suppression of unwanted qubit interactions for a parametric gate in a tunable superconducting circuit," Physical Review A **102**, 022619 (2020).

[12] R. Barends, C. Quintana, A. Petukhov, Y. Chen, D. Kafri, K. Kechedzhi, R. Collins, O. Naaman, S. Boixo, F. Arute, *et al.*, "Diabatic gates for frequency-tunable superconducting qubits," Physical review letters **123**, 210501 (2019).

[13] Y. Sung, L. Ding, J. Braumüller, A. Vepsäläinen, B. Kannan, M. Kjaergaard, A. Greene, G. O. Samach, C. McNally, D. Kim, *et al.*, "Realization of high-fidelity cz and z z-free iswap gates with a tunable coupler," Physical Review X **11**, 021058 (2021).
